# A forward-viewing endoscope and a rotatable sphincterotome successfully enabled biliary intervention with esophageal stenosis

**DOI:** 10.1055/a-2899-0828

**Published:** 2026-07-08

**Authors:** Tomohiro Ishii, Kazuya Sugimori, Arisa Omata, Hideyuki Anan, Takashi Kurosawa, Shin Maeda

**Affiliations:** 1 Department of Gastroenterology 89460 Saiseikai Yokohamashi Nanbu HospitalYokohamaKanagawaJapan; 2 Gastroenterology DivisionYokohama City University, School of MedicineYokohamaKanagawaJapan

Performing endoscopic retrograde cholangiopancreatography (ERCP) with a large-diameter side-viewing duodenoscope with esophageal stenosis might be difficult due to the narrowed lumen. In such situations, temporary esophageal stent placement or overtube use has been reported as an alternative approach. 1​ ​2​ If a side-viewing duodenoscope cannot pass through stenosis, ERCP using a forward-viewing endoscope might also be considered. However, this approach is scarcely reported except in patients with surgically altered stomach anatomy. 3​ ​4​ Recently, using sphincterotomes with high rotational and deflection capabilities has been reported. 5​ Herein, we report a successful ERCP with esophageal stenosis using a forward-viewing endoscope and a rotatable sphincterotome.

**Fig. 1:**
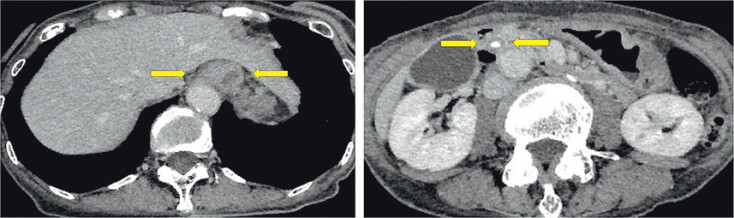
Portal phase of contrast-enhanced CT revealed wall thickening at the gastroesophageal junction (left). Contrast-enhanced CT showed a common bile duct stone (right). CT, computed tomography.

The patient was a 74-year-old woman scheduled to undergo surgery for gastroesophageal junction cancer. Due to liver dysfunction, abdominal computed tomographic scan and magnetic resonance cholangiopancreatography were performed, revealing a common bile duct stone ( **Figs.** 1 **and** 2 ). Since the intrahepatic bile ducts were narrowed, percutaneous intervention was difficult; therefore, we attempted ERCP. To evaluate the esophageal stenosis, we began with a forward-viewing endoscope (GIF-H290T; Olympus, Tokyo, Japan) prior to ERCP. Although a tumor narrowed the gastroesophageal junction, the endoscope was managed to pass through that section. We determined that inserting a large-diameter side-viewing endoscope would be difficult; therefore, we performed ERCP using the forward-viewing endoscope. Reaching the duodenum, the papilla of Vater was located at the edge of a large papillary diverticulum. Although the papilla could not be visualized en face, rotation and deflection of a rotatable sphincterotome (Engetsu; Kanekamedix, Osaka, Japan) enabled successful biliary cannulation ( Video 1 ). After endoscopic papillary balloon dilation, the stone was fragmented and removed. A biliary stent was placed for residual stones. Subsequently, we successfully removed the biliary stent and remaining stones. Endoscopic biliary intervention using a forward-viewing endoscope and a rotatable sphincterotome could be a feasible and effective approach in patients with esophageal stenosis.

**Fig. 2:**
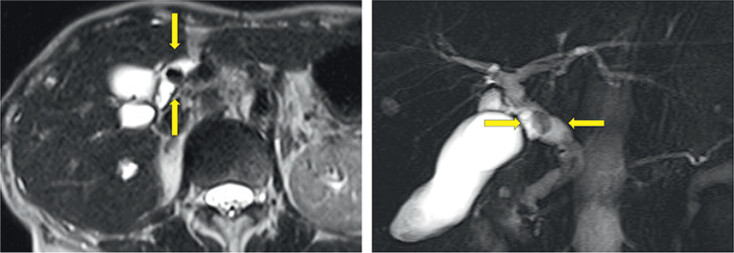
In an axial view, T2-weighted magnetic resonance imaging showed a common bile duct stone (left). Magnetic resonance cholangiopancreatography also revealed a common bile duct stone (right).

**Video 1** Rotation and deflection of a rotatable sphincterotome enabled successful biliary cannulation.


Endoscopy_UCTN_Code_TTT_1AR_2AK
